# Numerical simulation of the seismic response of corroded pipelines crossing faults

**DOI:** 10.1038/s41598-026-49476-1

**Published:** 2026-04-28

**Authors:** Ying Li, AoYing Zhang

**Affiliations:** 1 School of Earthquake Engineering and Building Safety, Institute of Disaster Prevention, San he, 065201 China; 2 Hebei Technology Innovation Center for Multi-Hazard Resilience and Emergency Handling of Engineering Structures, San he, 065201 China

**Keywords:** Buried pipeline, Internal corrosion, Normal fault, Local buckling, Stepped corrosion, Engineering, Materials science

## Abstract

Buried steel pipelines serve as core infrastructure for oil and gas transportation. Their corrosion-fault coupling failure under seismic fault activity poses a severe threat to energy security. The present study focuses on an X52 pipeline. The establishment of a finite element model of an internally corroded pipeline crossing a normal fault is based on the ABAQUS finite element software. By comparing the strain responses of corrosion-free and corrosion-defective pipelines, the failure mechanism under the coupled action of normal faults and corrosion is revealed. Through contrasting the failure modes and corrosion locations of reverse-faulted pipelines, the most vulnerable position of corrosion-affected pipelines under normal faulting is verified. Additionally, the study analyzes the influence of stepwise corrosion—characterized by the simultaneous evolution of three parameters: corrosion depth (h/t), corrosion width (w), and corrosion length (l)—under positive faulting on the strain behavior and failure mechanisms of pipelines. The results indicate that local corrosion defects significantly intensify the concentration of compressive strain in the pipeline, triggering local buckling, thereby weakening the pipeline’s overall deformation capacity and stability. Compared with tensile failure, normal fault action is more likely to cause compressive failure of the pipeline. The positioning of the corrosion defect at the peak compressive strain region on the pipe crown, consistent with the worst-case principle, can facilitate the accurate revelation of the pipeline failure mechanism under normal fault action. This, in turn, provides a basis for the optimisation of the pipeline’s anti-buckling design. Escalation in corrosion stage expedites the deterioration of the pipeline’s mechanical performance, evidenced by an earlier onset and more accelerated progression of local buckling, consequently diminishing the pipeline’s capacity to resist fault displacements. The present study reveals the critical influence of more realistic corrosion forms and fault coupling effects on pipeline failure behavior, providing theoretical support for the safety assessment and seismic design of corroded pipelines in practical engineering.

## Introduction

The issue of buried pipelines, which play a critical role in the transportation of oil and gas, is of particular concern when they traverse active fault zones, as they are susceptible to severe seismic safety threats. Permanent ground displacement associated with normal faulting has the potential to induce local buckling, tensile fracture, or compressive collapse of the pipeline. The presence of corrosion defects can further exacerbate the degradation of the pipeline’s mechanical performance. In the context of contemporary research endeavors, the failure mechanism that emerges from the interplay of normal faulting and corrosion remains to be fully elucidated. A conspicuous absence in the current research is a systematic analysis of the combined effects of corrosion location and fault displacement. General corrosion is characterized by uniform thinning of the pipe wall, which inevitably reduces the overall capacity of the pipeline. It has been extensively studied using cyclic loading models^1^. In contrast, pitting corrosion represents a more localized threat. Electrochemical studies indicate that both transient and steady-state pitting coexist, jointly governing the evolution of the corrosion process. Among these, pitting depth emerges as a critical factor influencing structural integrity degradation^2^. Stepwise corrosion bridges these two extremes, involving simultaneous evolution of corrosion depth, width, and length, thereby forming a progressive, multi-stage degradation process. Existing reviews indicate that numerical simulations predominantly employ simplified corrosion geometry models^3^. This study aims to address this gap by establishing a three-stage stepped corrosion model: a stepped corrosion process featuring the synchronous evolution of three parameters—corrosion depth, corrosion width, and corrosion length. This model realistically reflects the corrosion progression under normal faulting conditions.

A considerable body of research has been conducted by numerous scholars on the failure mechanisms of buried pipelines under the coupled action of normal faults and corrosion. Jin Liu et al.^4^ determined a reasonable range for the computational domain in buried pipeline-soil interaction analyses through parametric analysis, thereby providing an important basis for subsequent finite element modeling. Vazouras et al.^5^ developed a three-dimensional finite element model of a buried steel pipeline under strike-slip fault action. The model incorporated shell and solid elements, and the pipeline’s strain distribution was systematically analyzed. Xue Na et al.^6^ directed their attention to the failure mode of buried pipelines crossing normal faults, thereby elucidating the characteristics of compressive failure induced by normal fault action. Wei et al.^7^ conducted a systematic study of the mechanical behavior of buried pipelines under fault action. This study employed parametric analysis to investigate the influence of pipeline-soil interaction on pipeline deformation. In their study, Han et al.^8^ examined the response characteristics of pipelines under various fault types. Their findings indicated that transpressional reverse faults posed the most significant threat to pipelines. Additionally, under normal faulting conditions, compressive failure was observed to be more probable than tensile failure. Jalali et al.^9^ employed a combination of experimental methods and finite element analysis to investigate the impact of reverse faulting on continuous steel gas pipelines. This approach enabled the validation of the numerical model’s reliability. Cheng Xudong et al.^10^ investigated the impact of corrosion defects and examined the failure modes of corroded pipelines under strike-slip fault action. Their analysis revealed that the corrosion depth significantly exacerbates strain concentration. Zhang Rulin et al.^11^ developed a finite element model of a steel buried pipeline crossing a fault zone and, through numerical simulation, studied the pipeline’s strain response. They noted that the pipeline’s maximum strain points are located on both sides of the fault plane and that shallow burial and thick-walled design can improve seismic performance. In their study, Liu et al.^12^ employed numerical simulation to analyze the buckling failure mode of a high-grade X80 pipeline under reverse fault action, thereby providing a valuable reference for the seismic design of high-strength steel pipelines. Liu Ziliang et al.^13^ expanded the research to encompass the emerging field of energy, undertaking a comprehensive analysis of the distinctive failure behavior exhibited by buried hydrogen pipelines when subjected to strike-slip fault activity.

Data-driven structural assessment and damage identification methods demonstrate significant advantages in predicting the performance of complex engineering structures, offering new perspectives for failure analysis under multi-factor coupling effects such as corrosion. For instance, Zhang et al.^14^ proposed a framework integrating multi-source real data with machine learning to achieve high-precision prediction of weld fatigue life under conditions of small samples and high noise. Their approach—quantifying parameter uncertainty via variational Bayesian inference and enhancing key feature recognition through self-attention mechanisms—provides reference for analyzing the stochastic characteristics of mechanical properties in corroded pipelines. Chen et al.^15^ developed an interpretable Random Forest (RF) surrogate model. By generating stress intensity factor (SIF) datasets via finite element simulations, they efficiently predicted SIF and fatigue life for double-sided butt welds. SHAP analysis revealed stress and crack depth as core influencing factors, validating the feasibility of data-driven surrogate models to replace complex numerical simulations. Similar approaches can be extended to quantify the impact of pitting depth and uniform corrosion wall thickness on strain concentration in pipelines. Zhang et al.^16^ proposed a multi-stage image enhancement approach combined with an improved DeepLabV3 + lightweight model for underwater structural damage detection, enabling precise identification of defects such as cracks and exposed reinforcement. This provides technical insights for quantifying corrosion in pipelines under complex environments. These studies enhance structural information authenticity and model generalization through data-driven approaches, aligning with this research’s focus on analyzing step-type corrosion-fracture combination effects. Future work may integrate machine learning methods to establish rapid prediction models for corrosion-fracture coupled failure.

This study proposes solutions to the aforementioned issues. First, it compares the strain responses of pipelines with different corrosion locations (pipe crown and invert) under reverse and normal fault action. This comparison identifies the critical region for compressive failure under normal faulting. The study proposes placing the corrosion defect at the peak compressive strain region on the pipe crown to more accurately simulate the failure mechanism. Second, it designs a Third-Order Incremental Corrosion Model with Simultaneous Evolution of Three Parameters: Corrosion Depth (h/t), Corrosion Width (w), and Corrosion Length (l) to reveal the influence of simultaneous variations in three parameters—corrosion depth, width, and length on the pipeline’s ability to resist fault displacement. It also establishes a mapping relationship between corrosion stage and critical compressive strain and analyzed the pipeline’s seismic performance based on failure criteria and other factors. This approach provides a theoretical foundation for the seismic design and safety assessment of complex corroded pipelines under normal fault action.

## Numerical model establishment

### Geometry of undamaged and corroded models

In establishing the finite element model of a buried pipeline crossing a fault, it is essential to reasonably determine the model dimensions to accurately capture the pipeline-soil interaction and the large deformation response induced by fault slip. In accordance with the principles established by seismic design codes and the prevailing numerical simulation practices, the axial length of the pipeline within the area affected by the fault should be adequate to meet the minimum length requirement. The pipeline segment in proximity to the fault that may undergo significant deformation should have a minimum diameter of 60 times the pipe diameter. In this study, the total pipeline length is set to 62D = 32 m (where D is the pipe outer diameter) to ensure that the plastic deformation zone induced by fault action is covered.

**Fig. 1:**
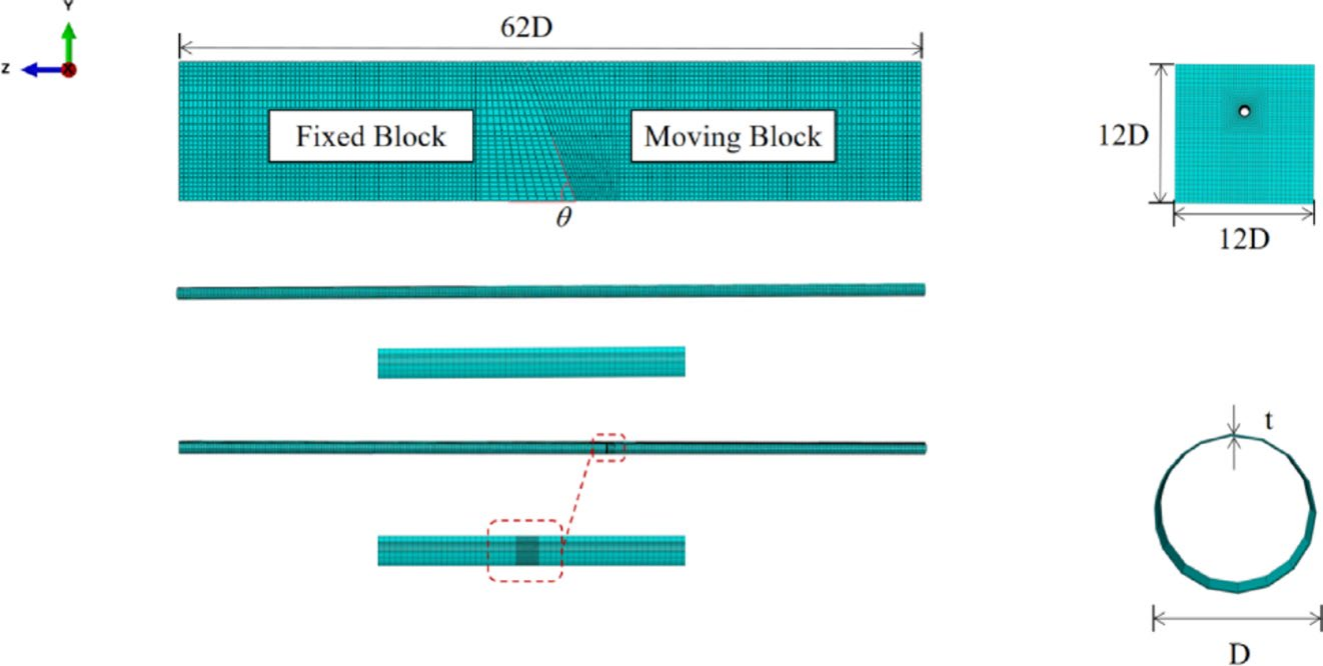
Finite Element Model Diagrams of Intact and Damaged Pipelines.

In order to eliminate boundary effects and fully reflect soil constraints, the soil domain has been set to the same length as the pipeline along the axial (Z) direction, 62D = 32 m. The dimensions of the soil domain perpendicular to the pipeline (horizontal X direction and vertical Y direction) have been set to 12D = 6 m, in order to meet the modeling requirements for three-dimensional soil stress transfer. The 3D finite element model depicted in Fig. 1, with the aforementioned dimensions, attains a sophisticated simulation of the coupled system comprising fault, pipeline, and soil.

### Soil mass and pipeline parameters

This study utilizes X52 steel pipe with key material properties: Elastic Modulus E1 = 210*GPa*; Poisson’s Ratio 0.3;${\sigma _1}=419MPa;{\varepsilon _1}=0.002;{\sigma _2}=570MPa;{\varepsilon _2}=0.054.$

The soil block surrounding the pipeline measures 32 m in length, with a width and height of 6 m. The soil in this area is classified as Malan loess. The Mohr-Coulomb (M-C) model has gained widespread acceptance due to its consistent alignment with experimental outcomes. The present study adopts the M-C constitutive model for the soil, a decision that is informed by the characteristics of the pipeline-fault-soil interaction. The adoption of the M-C model is based on the following considerations: parameters can be readily obtained through conventional geotechnical tests; it has been thoroughly validated in engineering practice with high experimental correlation; and its implementation in ABAQUS is well-established. However, it also has certain limitations: it cannot accurately predict volume changes caused by shear swelling/shear shrinkage under large strains, and it is unsuitable for cumulative deformation analysis under cyclic loading conditions. Since this study involves monotonic fault displacement, the impact of these limitations on the results is manageable. With respect to deformation behavior, the M-C model can be divided into two typical stages: an elastic stage and an elastoplastic stage. The model under consideration contains five key parameters: two parameters controlling elastic behavior (elastic modulus E and Poisson’s ratio ν) and three parameters controlling plastic behavior (cohesion c, friction angle φ, and dilatancy angle ψ). The relevant parameters are enumerated in Table 1.

**Table 1:** Soil Parameters Around Pipeline.

Physical Performance Indices	Numerical Values
Soil Bulk Density /(kg/m3)	1800
Elastic Modulus/MPa	10
Poisson’s Ratio	0.35
Cohesion/kPa	50
Friction Angle/°	20
Dilation Angle/°	0.1

### Contact modeling and loading

In order to simulate the response of the buried pipeline to normal fault action, a three-dimensional solid model of the pipeline and the faulted soil is constructed. This model utilizes C3D8R reduced-integration eight-node hexahedral solid elements. Corrosion is modeled by reducing the pipe wall thickness in uniform steps. This element type offers several key advantages in engineering applications, including high accuracy in capturing deformations, adaptability to large deformations, and efficient stress analysis. These properties enable it to accurately reflect the mechanical response of the pipeline under normal fault displacement.

The interaction between the pipeline and the soil is modeled through the separation of the contact behavior into normal and tangential components. In the context of normal behavior, a hard-contact criterion is adopted, thereby facilitating the transfer of compression and ensuring separation to prevent non-physical penetration. In the case of tangential behavior, a penalty friction model is implemented, with the interface friction characterized by friction coefficients. The friction coefficients for the pipeline-soil interface and soil-soil interface are taken as 0.5 and 0.3, respectively.

**Fig. 2:**
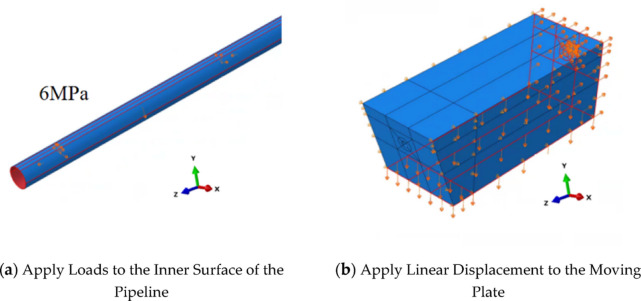
Loading Configurations on Pipeline Inner Surface and Moving Plate.

In the numerical simulation, a gravitational acceleration load of 9.8 m/s² is applied to the entire model in the vertically downward direction (i.e., along the negative Y-axis). The internal pressure of the pipeline is prescribed as 6 MPa as shown in Fig. 2. Utilizing the “angle-based selection” technique to ascertain the effective surface guarantees even and ongoing application of internal pressure to the pipe’s inner wall, facilitating accurate load simulation. No constraints are applied to the top surface of the upper soil block; normal constraints are applied to the sides of the soil blocks; the top of the lower soil block is set as a free surface, and the bottom is fully fixed; the sides of the model and the boundary far from the fault are applied with normal displacement constraints, allowing only tangential free deformation along these boundaries; the pipeline ends use free boundary conditions.

**Fig. 3:**
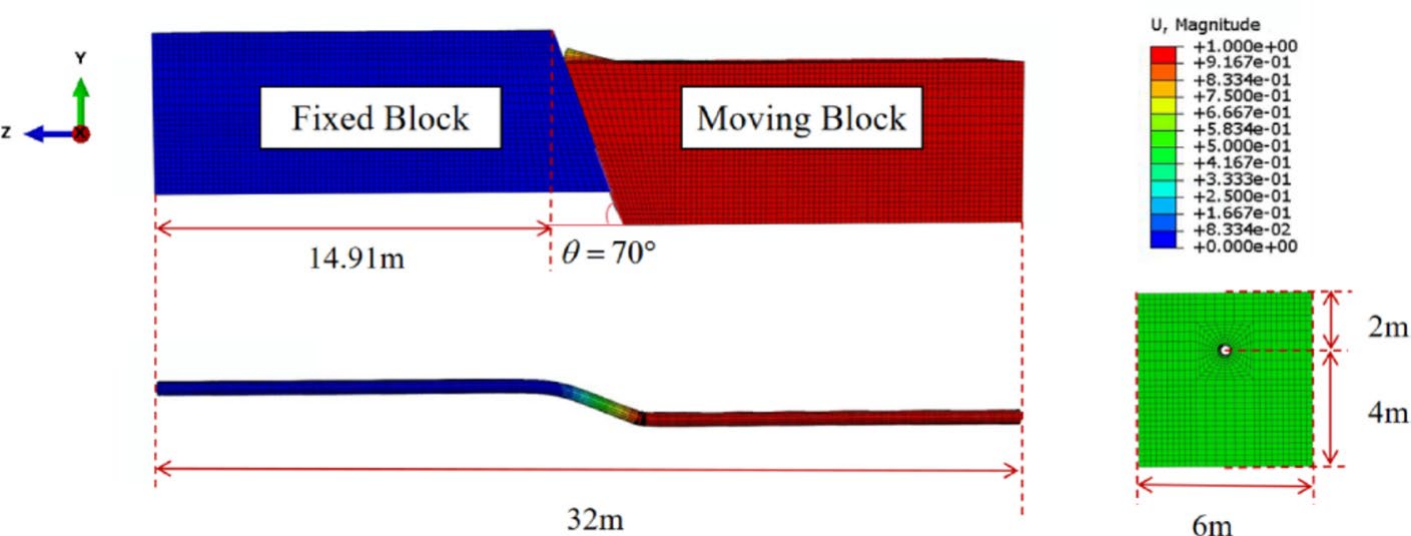
Soil-pipeline deformation response under 1 m fault displacement.

**Fig. 4:**
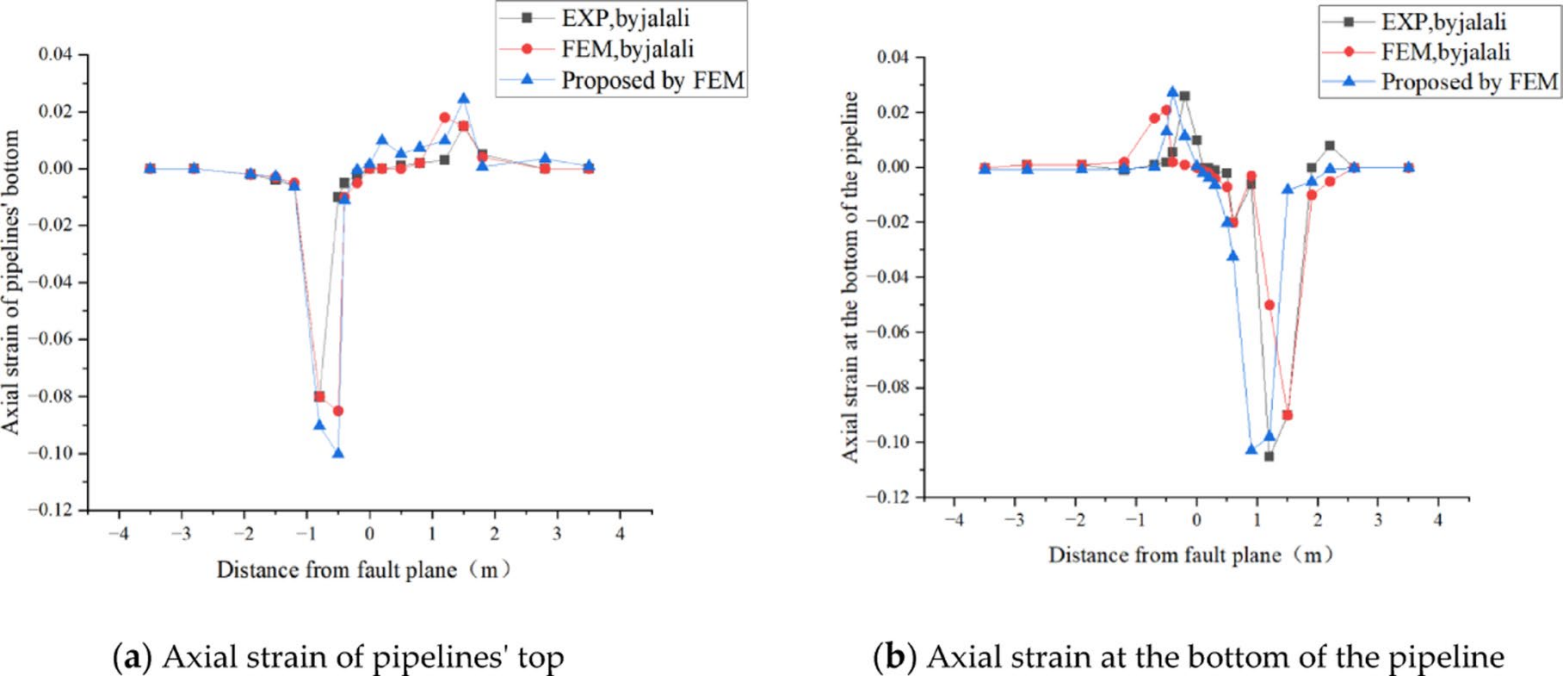
Comparison of Axial Strain in Pipelines.

The finite element analysis is performed in two steps. The first step involves the implementation of an in-situ stress equilibrium step, also known as static consolidation, which aims to establish a state of stress equilibrium between the surrounding soil and the pipeline. This step enables the consolidation of the soil. The second step involves the execution of an implicit dynamic analysis step, which simulates the potential fault slip. The fundamental reason for selecting the implicit dynamic step in this study lies in the fact that fault slip represents a quasi-static large-deformation process involving multiple nonlinearities (contact, material, geometry). The implicit dynamic analysis step simulates fault slip through an incremental loading method, accurately reflecting the dynamic influence of fault displacement on the pipeline. This approach effectively addresses the establishment of pipe-soil contact while ensuring computational stability and accuracy (Figs. 2, 3 and 4).

### Mesh discretization

In this study, all models utilize structured mesh discretization. In the vicinity of the pipeline-soil interface, the interaction leads to significant alterations in stress and strain, manifesting as pronounced gradients. A coarse mesh is inadequate for capturing the nonlinear variations present in such analyses, as it can result in inaccurate outcomes or even non-convergence. Conversely, refined meshes possess the capability to more accurately compute contact pressures, frictional stresses, and slip, thereby facilitating a more realistic representation of soil yielding, plastic zone development, and damage evolution. Consequently, the soil surrounding the pipe is subject to local refinement. Along the pipe’s circumference, local mesh seeds are configured to generate 24 divisions, with refinement applied to regions exhibiting significant deformation and areas of corrosion as shown in Fig. 3. Following a thorough computational process, satisfactory convergence was attained.

### Finite element model validation

The finite element model was validated using full-scale experimental data from Jalali et al.^9^ which served as a benchmark for the analysis. A corresponding numerical model of the pipeline-soil interaction was established based on their experimental (EXP) and finite element method (FEM) results^17-20^. Both the axial strains at the top and bottom of the pipeline within 4 m on each side of the fault plane were extracted and compared with the experimental data, showing good agreement as shown in Fig. 4. This finding serves to substantiate the rationality and precision of the finite element model^21^.

### Failure criteria for fault-crossing corroded pipelines

#### Strain-based failure criteria

According to GB/T 50,470 − 2017, “Code for Seismic Design of Oil and Gas Pipeline Engineering,” the design of buried steel pipelines under normal fault action should adopt a strain-based criterion to control the plastic deformation induced by fault displacement^22,23^. This method evaluates the strain capacity in three limit states—tension, compression, and local buckling—as failure criteria, prioritizing the pipeline’s overall stability within permissible deformation^24,25^. This approach replaces the conventional stress-based design’s rigid elastic-limit constraints, thereby enhancing its capacity to accurately reflect the displacement-driven characteristics inherent in fault loading.

### Tensile failure

The tensile failure design criteria under the strain-based approach are compared in Table 2. When the axial tensile strain in the pipeline wall induced by fault displacement exceeds the ultimate tensile strain capacity, the pipeline will rupture. The establishment of strain limits is predicated on a comprehensive evaluation of the weakening effects of key factors, as delineated in current codes^26^. Such factors include wall thinning resulting from corrosion defects, the softening of weld heat-affected zones, and additional thermal stresses, all of which exert influence on the pipeline’s mechanical properties. The evaluation process ultimately yields strain limit values that are recommended for use, with these values being derived from full-scale tests and fracture mechanics analysis.

**Table 2:** Ultimate Tensile Strain Under Different Standards and Specifications.

Standards and Specifications	Ultimate Tensile Strain/%
CAS-Z2662-23(2023)	2.5
DNV-ST-F101(2021)	2.0
GB/T 9711 − 2023(2023)	2.0

### Local buckling

According to standard CAS-Z2662-23 (2023)^27^, the critical longitudinal compressive strain for local buckling can be expressed as:$\varepsilon _{c}^{{crit}}$

1 $$\varepsilon_c^{crit} = 0.5\frac{t}{D} - 0.0025 + 3000\left( {\frac{PD}{{2tE_s }}} \right)^2 for\frac{PD}{{2tF_y }}<0.4$$

2 $$\varepsilon_c^{crit} = 0.5\frac{t}{D} - 0.0025 + 3000\left( {\frac{0.4F_y }{{E_s }}} \right)^2 for\frac{PD}{{2tF_y }}\geqslant0.4$$

In the geometric:

$\varepsilon _{c}^{{crit}}$—Critical Compressive Strain.

t—Wall Thickness, mm.

D—Outside Diameter, mm.

P—Internal Pressure, Mpa.

E_s_—Elastic Modulus, 207000Mpa.

F_y_—Minimum Specified Yield Strength, Mpa.

Following a thorough examination of the pertinent data and the subsequent integration of the relevant parameters, the ultimate value of the critical longitudinal compressive strain for local buckling has been determined to be 0.00535.

### Ovalization

In order to avert the premature failure of the cross-section (excessive ovalization) that would impede the pipeline’s functionality under normal operating conditions, it is imperative to curtail the degree of pipeline ovalization in accordance with the following stipulations, with the limit value designated as 0.03 in accordance with CAS-Z2662-23 (2023).: $\Delta _{\theta }^{{crit}}$.

3 $${\Delta _\theta } \leqslant \Delta _{\theta }^{{crit}}$$

In the geometric:

$\Delta _{\theta }^{{crit}}$—Critical Ovalization Deformation.

$$\Delta _{\theta }^{{}}$$ = Ovalization Deformation

$$=2\left( {\frac{{{D_{\max}} - {D_{\min}}}}{{{D_{\max}}+{D_{\min}}}}} \right)$$

In the geometric:

D_max_—Maximum Outside Diameter, mm.

D_min_—Minimum Outside Diameter, mm.

## Results and discussion

### Comparison of strains between undamaged and locally corroded pipelines

In the ABAQUS simulation, a model was established in which the pipeline contains an internal corrosion defect of rectangular shape (circumferential width 45°, length L1, depth 30.7% of the wall thickness). In order to simulate the most unfavorable condition, this corrosion defect was located at the pipe crown, where the compressive strain is maximal. The numerical results indicate that under normal fault displacement, the undamaged pipeline accommodates the soil deformation through forced bending. As the fault displacement increases, the pipeline’s tensile and compressive strains gradually increase, but the maximum values remain below the material yield limit. The deformation mode of the pipeline remains stable, with no occurrence of local buckling or tensile necking. The findings indicate that within the range of displacements examined, the pipeline effectively absorbs the crustal deformation energy through plastic deformation. This process ensures the structural integrity and stability of the pipeline, thereby facilitating continuous safe operation.

**Fig. 5:**
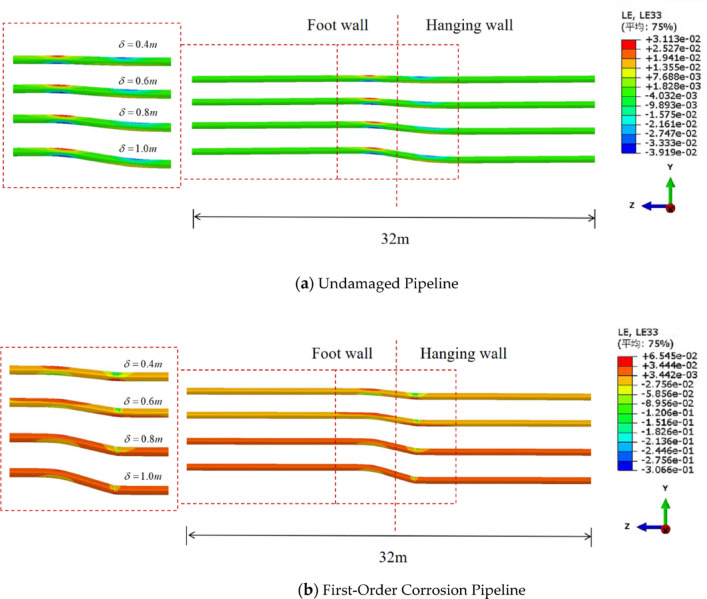
Stress Distribution Map of Pipeline Crossing Normal Fault.

In comparison with an undamaged pipeline, a corroded pipeline demonstrates heightened compressive strain in the defective region. Concurrently, the tensile strain at the pipe invert beneath the defective region also exhibits an increase. The corrosion defect weakens the stability of the pipe wall, resulting in a significantly greater increase in compressive strain compared to tensile strain. This leads to the failure of the structure by local buckling well before reaching the tensile failure limit. The strain contour plots of the pipeline crossing a normal fault are displayed in Fig. 5. In this figure, (a) corresponds to the undamaged pipeline and (b) corresponds to the pipeline with a first-stage corrosion defect, where δ is the fault displacement.

### Effect of different corrosion locations (at regions of peak strain on the pipe crown)

During the configuration of corroded pipeline models, based on prior studies of buried pipeline behavior under reverse faulting, the corrosion position was initially established at the pipe crown compressive strain peak region to more accurately simulate realistic conditions under fault action. Initially, a pipeline with corrosion at the pipe crown compressive strain peak region was modeled for a reverse fault scenario. Subsequently, a model was constructed to illustrate the interaction of pipelines that traverse a standard geological fault. The model incorporated corrosion in the pipe crown, tensile strain peak, and compressive strain peak regions, respectively. As fault displacement increased, a comparison was made of the peak axial strains of these pipelines at the corrosion locations.

**Fig. 6:**
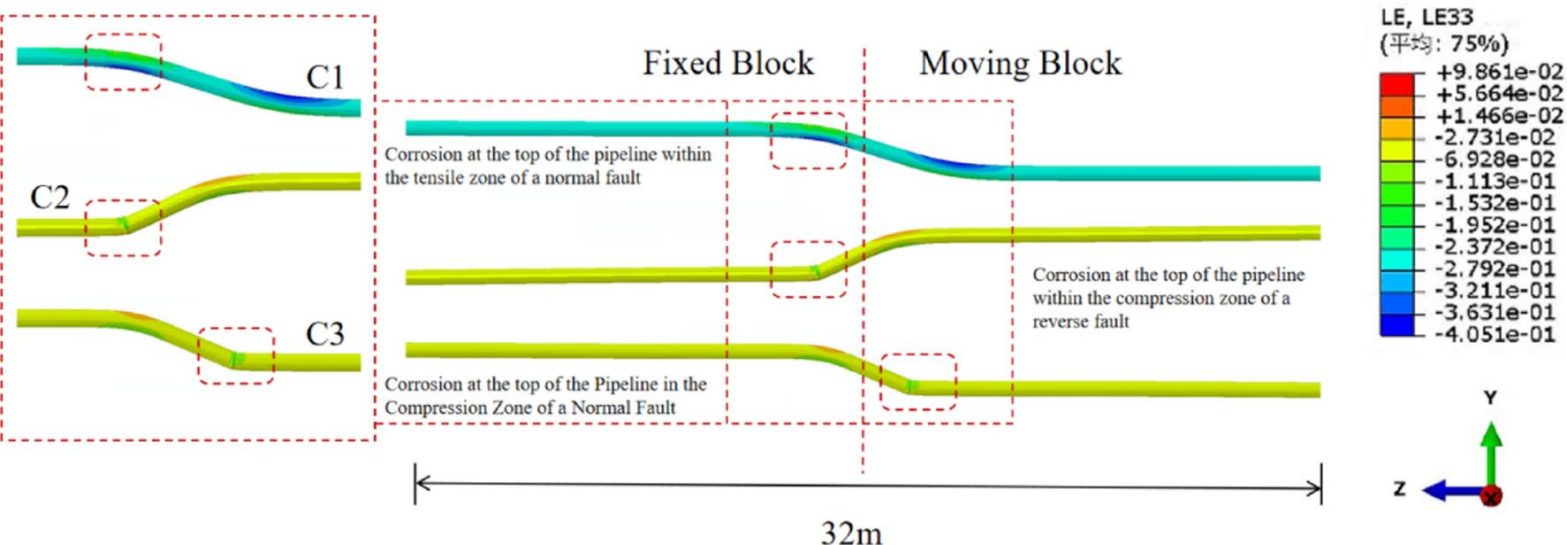
Strain Cloud Diagrams Under Different Fault Types and Corrosion Locations.

For these pipelines, models were constructed for both normal and reverse fault action, with the corrosion region situated at the locations of maximum axial strain (tensile or compressive) at the pipe crown. The comparison reveals that under normal fault action, the pipeline is less likely to experience tensile failure and is more prone to compressive local buckling. The strain contour maps presented in Fig. 6 (from top to bottom) illustrate the effect of a given fault displacement. Correspondingly, C1 corresponds to a normal fault with corrosion at the pipe crown tensile strain peak region; C2 corresponds to a reverse fault with corrosion at the pipe crown compressive strain peak region; and C3 corresponds to a normal fault with corrosion at the pipe crown compressive strain peak region.

**Fig. 7:**
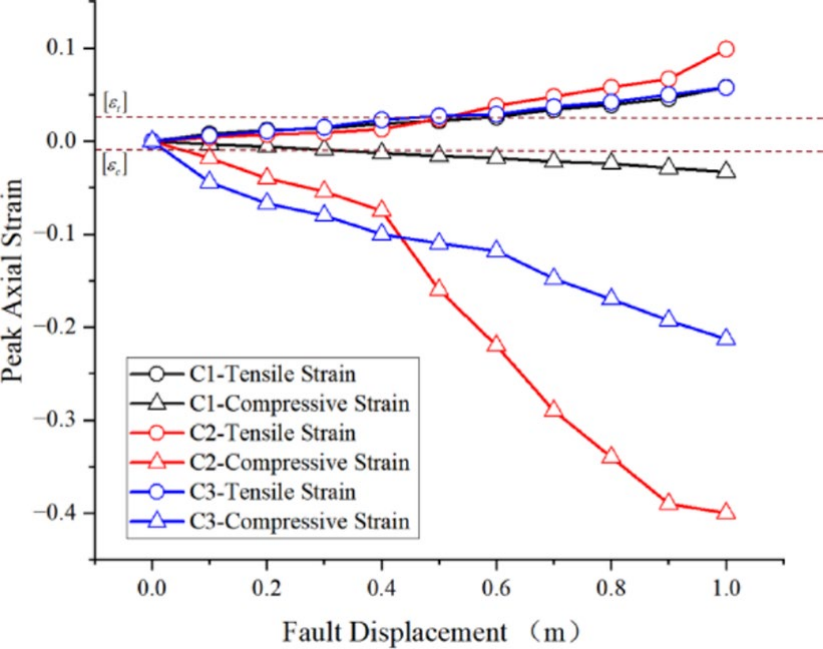
Peak Axial Strain Response to Fault Displacement Under Different Corrosion Scenarios.

Figure 7 presents the peak axial strain, expressed as the element-averaged value over the peak axial strain region, as a function of increasing fault-offset depth for the three previously mentioned corrosion scenarios. In this particular plot, both C2 and C3 demonstrate significantly higher compressive strains in comparison to C1, while the tensile strain of C2 exhibits a slight increase over that of C1 and C3. As fault displacement continues to increase, the compressive strain of C2 experiences a marked rise after a displacement of 0.4 m. Based on the data exceeding the strain limit value in this figure, combined with the strain contour plot in Fig. 6, it is determined that, for a given fault displacement, the reverse-fault scenario with corrosion at the crown compressive strain peak (C2) exhibits a heightened propensity for local buckling failure compared to the normal-fault cases with corrosion at the crown tensile (C1) or compressive (C3) strain peak regions.

### Effect of variation in corrosion stages on pipeline failure modes

**Fig. 8:**
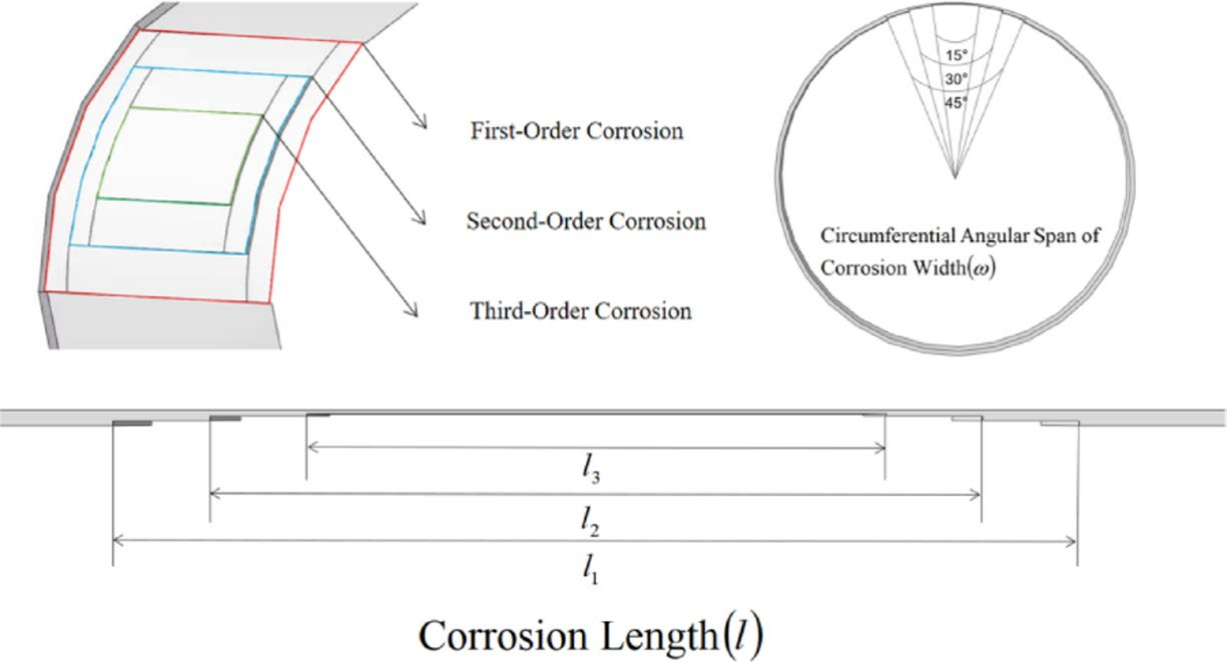
Detailed Schematic Diagram of Third-Order Corrosion Equivalence.

The corrosion model employed in this study deviates from the conventional rectangular pit model by incorporating a more authentic, stepped three-stage corrosion pattern, wherein the pit depth undergoes a successive increase. The initial stage of corrosion is characterized by a rectangular pit with a circumferential width of 45°, a length of L1, and a depth that is 30.7% of the wall thickness, denoted as D1. The second stage of corrosion introduces a pit with a width of 30°, a length of L2, and a depth of 2 mm, resulting in corrosion depths of 30.7% and 61.5%, denoted as D2. The third stage of corrosion introduces an additional pit at the center of the second stage, with dimensions of 15° in width, 3 mm in length, and 1 mm in depth. This results in final corrosion depths of 30.7%, 61.5%, and 76.9%, respectively. The maximum corrosion depths for the second and third stages are thus 61.5% and 76.9%, respectively, with D3 representing the Third-Order Corrosion. A comprehensive schematic illustrating the three-stage corrosion process is presented in Fig. 8. In order to analyze the pipeline failure mode under normal fault action, the corrosion location is set at the pipe crown compressive strain peak region to more accurately simulate local buckling under normal fault action.

**Fig. 9:**
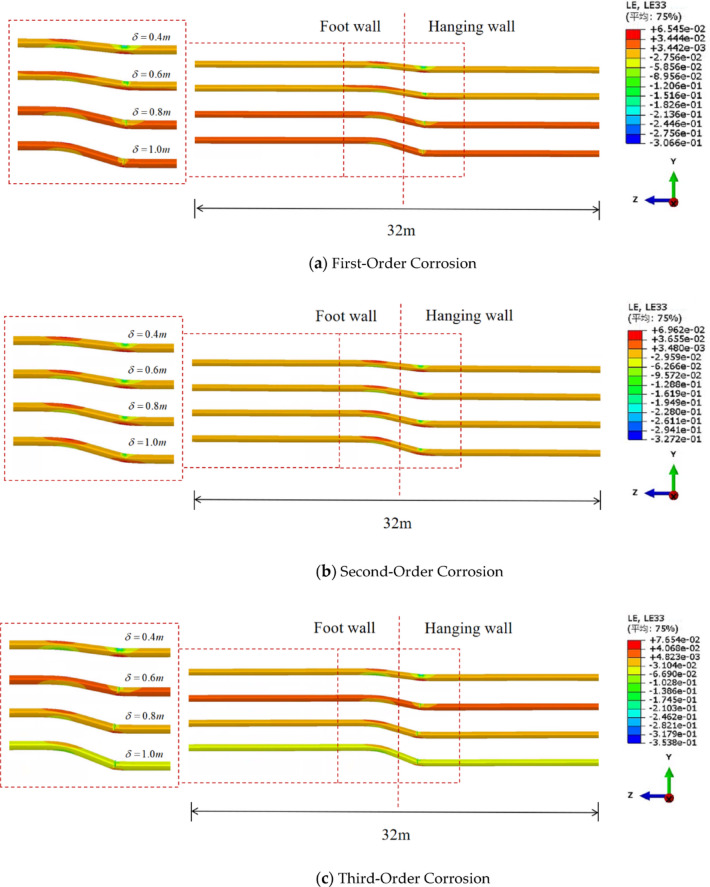
Strain Cloud Diagrams Under Different Corrosion Orders.

**Fig. 10:**
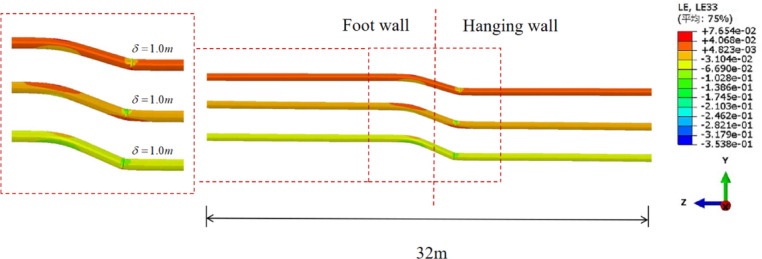
Strain Contour Plots of Three-Order Differently Corroded Pipelines Under 1.0 m Fault Displacement.

As illustrated in Figs. 9 (a) through (c), the strain contours are demonstrated for the initial, secondary, and tertiary stages of corrosion under various fault displacements. It has been observed that the onset of local buckling occurs earlier as the corrosion stage increases. A comparison of the strain contour maps at a fault displacement of 1.0 m reveals that local buckling becomes more pronounced and the failure becomes more severe with increasing corrosion stage. The comparative strain contour images at 1.0 m displacement are illustrated in Fig. 10.

**Fig. 11:**
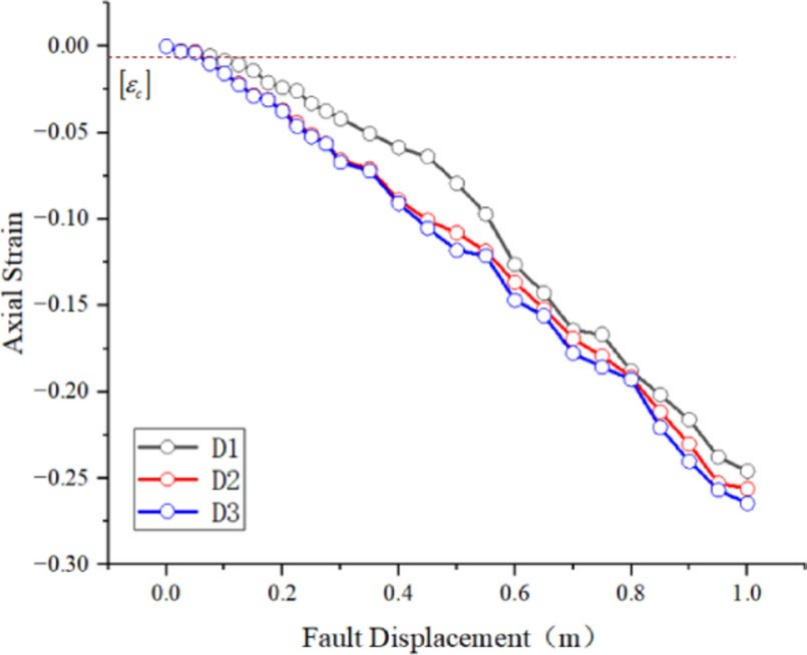
Peak Axial Strain Response to Fault Displacement Under Different Corrosion Scenarios.

As the fault displacement increases continuously, the axial compressive strain of the corroded pipeline also gradually increases. The primary distinction between the initial-stage and third-stage corrosion is the continuous escalation in corrosion depth. During the fault-crossing process, prior to reaching the critical compressive strain, pipelines with varying degrees of corrosion exhibit remarkably similar strain trends, thereby maintaining the established pattern that greater corrosion depth invariably results in higher axial strain. Subsequent to surpassing the critical compressive strain threshold, an increase in fault displacement is observed for varying corrosion depths. Consequently, the strains undergo fluctuations while maintaining the trend that an escalation in fault displacement or corrosion depth results in augmented axial strains as shown in (Fig. 11).

## Conclusions

This study utilizes the finite element software ABAQUS to establish a soil–pipe interaction model. Consider the performance differences between undamaged and third-order corroded pipelines. By analyzing both reverse fault and normal fault positions in combination with different corrosion locations, jointly infer the performance analysis and failure modes of buried steel pipelines under normal fault conditions. On this basis, the failure modes of pipelines with stepped corrosion at different corrosion stages are further investigated. Stepped corrosion is driven by the simultaneous evolution of three parameters: corrosion depth (h/t), corrosion width (w), and corrosion length (l). The purpose of this investigation is to more realistically capture the damage induced by the actual corrosion process.

- A comparison of finite element models indicates that placing a corrosion defect at the location of maximum compressive strain on the pipe crown significantly deteriorates the pipeline’s mechanical performance under fault action. In comparison with an undamaged pipeline, a corroded pipeline demonstrates a considerably elevated compressive strain in the defective region, eventually leading to local buckling failure in the corroded region. It is evident that localized corrosion poses a critical hidden hazard to the buckling resistance of buried pipelines subjected to fault movement. Therefore, in pipeline safety assessments, the prioritization of inspection focus areas, and the optimization of fault-resistant designs, the presence and impact of corrosion defects must be given high priority. This holds significant engineering guidance for enhancing the operational safety of buried pipelines in active fault zones.
- A simulation analysis was conducted to investigate the effects of corrosion positions on pipe crown tensile and compressive strain peak regions under both reverse and normal fault action. The analysis revealed that, under normal faulting conditions, the pipeline is more prone to compressive local buckling failure compared to tensile failure. The study indicates that by placing the corrosion defect at the pipe crown compressive strain peak region, the pipeline failure mode under normal fault action can be more accurately simulated. This provides direct guidance for optimizing pipe buckling resistance design in engineering projects. In practical applications, it is recommended to prioritize corrosion protection and monitoring in areas with concentrated stress concentrations at the pipe top, thereby preventing cascading failures triggered by localized buckling.
- Under normal fault action, an increase in the number of corrosion stages in the stepped corrosion model significantly exacerbates the degradation of the buried pipeline’s mechanical performance. The failure mode is characterized by earlier initiation and more severe development of local buckling. The synchronous evolution of corrosion depth, width, and length accelerates compressive strain concentration, causing pipelines to lose load-bearing capacity under smaller fault displacements. This finding offers new insights for improving current safety assessment methods based on single corrosion parameters. In engineering practice, multi-parameter coupled corrosion damage models should be established to more accurately reflect the mechanical behavior of corroded pipelines under dynamic fault action.

In conclusion, the modeling framework proposed in this study integrates soil-pipe interaction, multi-stage corrosion geometric characteristics, and fault dislocation parameters. It provides valuable data support for predicting local buckling and failure risks of buried corroded pipelines in fault zones. Future research could focus on sensitivity analyses of corrosion depth and fault displacement to provide insights for anti-buckling wall thickening thresholds and design criteria for truncated shear connectors.

## Data Availability

The datasets generated and analyzed during the current study are available from the corresponding author on reasonable request.

## Code availability

Our study utilized the commercial finite element software ABAQUS for all simulation processes. No custom code or algorithms were developed for this study. For any correspondence regarding the software model or requests for materials, please contact the corresponding author, A.Y.Z.
